# Finned Tubular Air Gap Membrane Distillation

**DOI:** 10.3390/membranes13050498

**Published:** 2023-05-08

**Authors:** Zhiqiang Wu, Fei Guo

**Affiliations:** School of Energy and Power Engineering, Dalian University of Technology, No. 2 Linggong Road, Dalian 116024, China

**Keywords:** finned tubular air gap membrane distillation, air gap structure, finned tube, transmembrane flux, air cooling

## Abstract

Finned tubular air gap membrane distillation is a new membrane distillation method, and its functional performance, characterization parameters, finned tube structures, and other studies have clear academic and practical application value. Therefore, the tubular air gap membrane distillation experiment modules composed of PTFE membrane and finned tubes were constructed in this work, and three representative air gap structures, including tapered finned tube, flat finned tube, and expanded finned tube, were designed. Membrane distillation experiments were carried out in the form of water cooling and air cooling, and the influences of air gap structures, temperature, concentration, and flow rate on the transmembrane flux were analyzed. The good water-treatment ability of the finned tubular air gap membrane distillation model and the applicability of air cooling for the finned tubular air gap membrane distillation structure were verified. The membrane distillation test results show that with the tapered finned tubular air gap structure, the finned tubular air gap membrane distillation has the best performance. The maximum transmembrane flux of the finned tubular air gap membrane distillation could reach 16.3 kg/m^2^/h. Strengthening the convection heat transfer between air and fin tube could increase the transmembrane flux and improve the efficiency coefficient. The efficiency coefficient (*σ*) could reach 0.19 under the condition of air cooling. Compared with the conventional air gap membrane distillation configuration, air cooling configuration for air gap membrane distillation is an effective way to simplify the system design and offers a potential way for the practical applications of membrane distillation on an industrial scale.

## 1. Introduction

Membrane distillation is a heat-driven membrane separation process where volatile molecules on the feed side can pass through the pores of a porous hydrophobic membrane, while nonvolatile molecules such as liquids and inorganic salts cannot. The molecules that pass through the membrane pores are then condensed on the cooling side [1,2,3]. Among the four basic configurations of membrane distillation, air gap membrane distillation is the one in which the hot feed contacts the membrane directly while the air gap separates the membrane from the condensation surface. This configuration reduces heat transfer losses and exhibits the highest thermal efficiency [4,5].

The structure of the feed side and the air gap will affect the performance of air gap membrane distillation. The addition of Λ-Ribs carbon-fiber open slots on the feed side can increase the transmembrane flux [6]. The thickness and structure of the air gap have a significant impact on the performance of air gap membrane distillation. A thinner air gap leads to a higher transmembrane flux [7]. To support thin porous hydrophobic membranes, maintain their flatness, and avoid mechanical stability limitations, support fillers are typically used in the air gap [8,9,10]. The use of fillers such as steel balls or sand can increase the transmembrane flux of membrane distillation [11,12,13,14]. Some researchers have found that adding fins to the condensation plate can increase the transmembrane flux, with square fins producing a higher transmembrane flux than circular or triangular fins [15]. Using the horizontal copper tubes to replace the common flat coolant plate could also enhance the membrane distillation performance [16]. Compared with the conventional air gap membrane distillation configuration (water cooling) is an effective way to simplify the system design and offers a potential way for the practical applications of membrane distillation on an industrial scale. Using air cooling in plate and frame air gap membrane distillation, the effect of water cooling can be achieved by forcing convection and increasing the heat transfer area [17,18].

Membrane distillation is a nonisothermal process, and the design of the configuration needs to consider flow conditions, packing density, heat recovery function, and thermal stability. Plate and frame membrane distillation components have the highest transmembrane flux but the lowest membrane surface area-to-volume ratio. To address the packing density issue, configurations such as hollow fiber, ceramic tube, and tubular membrane distillation can be used [19,20,21]. Among them, the tubular membrane component has a high packing density and large tube span, effectively solving membrane fouling problems and reducing polarization phenomena [22,23,24]. The finned tubular air gap membrane distillation configuration proposed by Cheng et al. combines the characteristics of air gap membrane distillation and tubular membrane distillation. By changing the air gap depth, quantity, and structure of the finned tubes, the transmembrane flux of the membrane distillation process can be adjusted [25]. The cylindrical and spiral air gap membrane distillation configurations also have good vapor condensation effects [26,27].

This paper focuses on the finned tubular air gap membrane distillation configuration and analyzes its mass transfer coefficient (*B*) and efficiency coefficient (*σ*). In particular, the study analyzes the influence of the structure of finned tubular (tapered finned tubular, flat finned tubular, and expanded finned tubular) on the air gap membrane distillation performance. The study also investigates the effect of the hot feed temperature, concentration, and flow rate on the mass transfer capability of the finned tubular air gap membrane distillation configuration. The impact of air cooling on finned tubular air gap membrane distillation applicability is also investigated using the membrane distillation tests. Furthermore, the study analyzes the effect of air temperature and condensation tube wall structure on the efficiency coefficient of the membrane distillation process. The membrane distillation test results show that the finned tubular air gap membrane distillation performance could be enhanced by optimizing the finned tubular structure. The maximum transmembrane flux of the finned tubular air gap membrane distillation could reach 16.3 kg/m^2^/h. Strengthening the convection heat transfer between air and the finned tube could increase the transmembrane flux and improve the efficiency coefficient.

## 2. Experimental

### 2.1. Membranes

**Figure 1:**
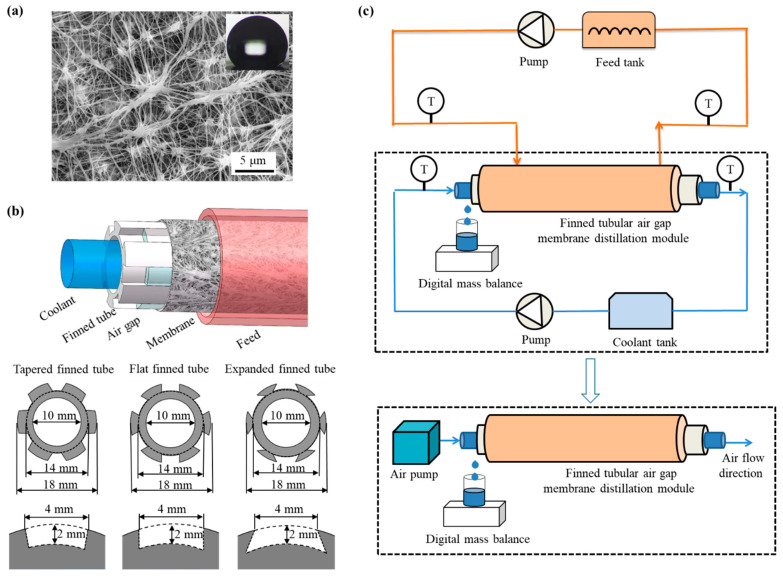
Parameters of the test membrane and the finned tubular air gap membrane distillation module used in this work. (**a**) The micromorphology of the test PTFE membrane characterized by SEM. (**b**) Schematic diagram of the finned tubular air gap membrane distillation module. (**c**) Schematic diagrams of finned tubular air gap membrane distillation tests.

Commercial polytetrafluoroethylene (PTFE) membranes (Membrane Solutions, LLC, Auburn, WA, USA) were used in finned tubular air gap membrane distillation tests. The membrane morphology was tested by scanning electron microscope (SEM) (QUANTA 450, FEI, Hillsboro, OR, USA), as shown in Figure 1a. The porosity of membranes was measured by the gravimetric method [28]. The thickness of the membrane was the average value of six measurements by micrometer (211-101F, Guilin Guanglu Measuring Instrument Co., Ltd., Guilin, China). The contact angle was the tentacle of the pure water (2.5 µL) on the membrane. The physical parameters of the membrane are shown in Table 1.

### 2.2. Finned Tubular Air Gap Membrane Distillation Module

The membrane assembly is composed of membrane and aluminum hollow finned tubes, as shown in Figure 1b. The feed is introduced from the outside of the membrane layer, and the coolant passes through the condensing tube in a concurrent manner. The gap is the space between the finned tube and the membrane, and six gaps are radially arranged along the finned tube. The grooves on the outer surface of the fins provide support for the membrane and also serve as air gaps in the configuration of the membrane distillation. The inclination angle between the module and the horizontal line was kept at 10 ± 1° to ensure that the condensate flowed out of the air gap in time. The parameters of the finned tubes are shown in Table 2.

### 2.3. Finned Tubular Air Gap Membrane Distillation Test

The experimental setup is shown in Figure 1c and consists of three main components: a feed circulation system, a finned tubular gap membrane distillation unit, and a cooling water circulation system. The feed (sodium chloride (NaCl) aqueous solution) is heated by a water bath (HH-2, Changzhou Zhiborui Instrument Manufacturing Co., Ltd., Changzhou, China) and transported to the finned tubular gap membrane distillation unit via a peristaltic pump (LM60A-YZ1515X-3B, Nanjing Runze Fluid Control Equipment Co., Ltd., Nanjing, China). The cooled feed is then returned to the feed tank. The coolant, with a temperature of 24 ± 1 °C and a flow rate of 0.27 m/s, is circulated from the cooling water tank to the finned tubular gap membrane distillation unit for heat exchange and then returned to the coolant tank. The weight of the beaker was recorded every minute using an electronic balance (BS-600+, Shanghai Friendship Scale Co., Ltd., Shanghai, China), and the temperature was recorded using a K-type thermocouple. To achieve a stable temperature condition, the experiment typically ran for 30 min before data collection began, and measurement data were recorded over a period of 1 h. The uncertainties of the instruments used in the experiment are shown in Table 3. 

In membrane distillation, coolant cooling requires a circulating system with cooling medium and an additional heat transfer cycle, which increases the complexity of membrane distillation systems. Using air cooling, a membrane distillation system requires only the introduction of a low-energy air pump (SC-60, Ningbo Sayer Electric Co., Ltd., Ningbo China), making the membrane distillation system more energy-efficient and compact. The air cooling device replaces the cooling water circulation system with a circulating air pump to achieve air flow in the condensation tube.

### 2.4. Mass Transfer for Membrane Distillation

The transmembrane flux is one of the parameters reflecting the mass transfer efficiency of membrane distillation. In the experiment, the transmembrane flux in the process of membrane distillation is calculated through the mass of collected condensate water, which can be calculated by the following formula:

(1) $$J=\frac{m}{At}$$

where *J* represents the transmembrane flux in kg/m^2^/h, *m* is the mass of the condensate collected during in kg, *t* is the predetermined time of the experimental process in h, and *A* is the size of the test PTFE membrane in m^2^. The accuracy of the transmembrane flux is evaluated by the standard deviation.

During the membrane distillation process, mass transfer across the porous membrane occurs through diffusion of water molecules in microchannels driven by the vapor pressure gradient. It depends on pore size, porosity, curvature, and collision behavior. Performance conditions of the membrane distillation device, such as temperature and concentration of the hot feed solution, membrane, and air gap parameters, affect the mass transfer capability of the membrane distillation process. The cooling conditions on the cold side can be characterized by the efficiency coefficient (*σ*) of the membrane distillation process, which can be described by Equation (2) to show the performance of the membrane distillation process [18,29]:

(2) $$J=B\sigma \left(P_{f}-P_{c}\right)$$

where *B* value is the total mass transfer coefficient including the mass transfer of water vapor in the feed side, the membrane pores, and the air gap. It is related to the configuration of membrane distillation module and the parameters of the membrane, with units of kg/m^2^/Pa/s. In the experiment, only one experiment is needed to calculate the *B* value, which can predict the trend of transmembrane flux during air gap membrane distillation process [29]. *σ* is the efficiency coefficient used to characterize the cooling capacity on the cooling side. The value of *σ* ranges from 0 to 1. It is approximately equal to 1 in a water-cooled membrane distillation. When air cooling is used in membrane distillation, the temperature difference between air temperature and condensing plate temperature is large. The *σ* value is used to correct the temperature of the condensing plate so that the transmembrane flux of the membrane distillation module can be predicted directly by calculating the saturated vapor pressure from the air temperature [18]. *P_f_* and *P_c_* are the saturated vapor pressures on the feed side and the cooling side, respectively, with units of Pa. The average temperature values on both sides of the feed can be calculated using the Antoine equation. These performance parameters are influenced by various factors, such as the parameters of the membrane, the air gap, and the temperature and concentration of the feed solution.

In air gap membrane distillation, there is no mixing between the permeate water and coolant, so the salt rejection (*R*) performance of it is simply calculated based on the concentrations of feed and permeate water:

(3) $$R=1-\frac{c_{p}}{c_{f}}$$

where *c_p_* and *c_f_* are the concentrations of the permeate water and feed, respectively.

## 3. Results and Discussion

### 3.1. Effect of Air Gap Structures

**Figure 2:**
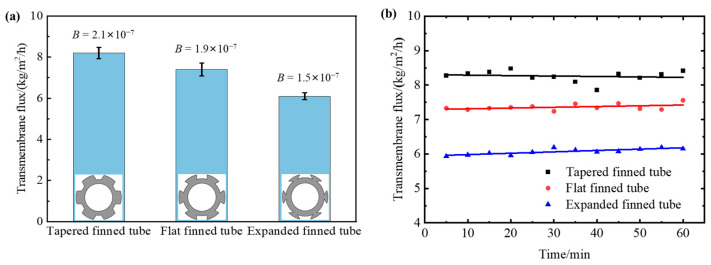
Functional performance of finned tubular air gap membrane distillation configurations with different finned tubular structures. (**a**) *B* value and transmembrane flux, (**b**) transmembrane flux along time. The concentration of the feed is 50 g/kg. The temperature of the feed is 54 ± 1 °C. The feed flow rate is 100 mL/min.

The membrane distillation process is cooled by water; the cooling is considered sufficient with the *σ* value of 1. The obtained transmembrane flux and *B* value are shown in Figure 2a, with the gradually expanding air gap structure having the lowest transmembrane flux, and the gradually shrinking air gap structure having the highest transmembrane flux. The salt rejections of all the membrane distillation tests are larger than 99.9%. The three configurations showed stable transmembrane mass transfer ability during the membrane distillation tests. Figure 2b shows the transmembrane flux along time.

The finned tube structure causes the condensed vapor to experience a small centrifugal effect as it passes downward through the fins, which helps produce additional inertial suction force on the vapor from outside the membrane, allowing the vapor to diffuse rapidly through the membrane and further promote surface vaporization [27]. As the tapering structure’s volume decreases in the direction of steam entry, the gas is compressed and easily condenses into droplets, resulting in the largest transmembrane flux and *B* value for this structure. Due to the PTFE hydrophobic membrane’s susceptibility to stretching, it is squeezed into the gas gap by the impact of the hot feed liquid. Since the shrinking structure has a smaller internal space, the gas gap thickness is reduced to some extent, reducing the heat transfer resistance and increasing the transmembrane flux and *B* value of membrane distillation. The shrinking structure may also bring benefits because the condensate cannot be immediately drained away [11,30]. The shrinking structure not only reduces the air gap thickness, but the device is closer to the water gap membrane distillation, which has a higher transmembrane flux than air gap membrane distillation. Therefore, the shrinking air gap structure performs better than the expanding finned tube for finned tubular air gap membrane distillation, and the benefits of the shrinking structure outweigh those of the expanding structure with a larger condensation area.

Table 4 shows a comparative study for air gap membrane distillation by considering different modules. It is obvious that the performance of the present finned tubular air gap membrane distillation module is in the range of the literature results and presents a viable choice with design flexibility.

### 3.2. Effect of Feed Parameters

**Figure 3:**
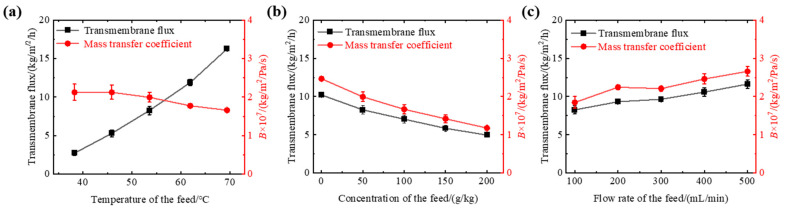
Functional performance of membrane distillation process under different feed conditions. (**a**) Transmembrane flux and *B* value of the tapered finned tubular air gap membrane distillation process at different feed temperatures. The concentration of the feed is 50 g/kg. The feed flow rate is 100 mL/min. (**b**) Transmembrane flux and *B* value of the tapered finned tubular air gap membrane distillation process at different concentrations of the feed. The temperature of the feed is 54 ± 1 °C. The feed flow rate is 100 mL/min. (**c**) Transmembrane flux and *B* value of the tapered finned tubular air gap membrane distillation process at different flow rates of the feed. The concentration of the feed is 50 g/kg. The temperature of the feed is 54 ± 1 °C.

Experimental results shown in Figure 3a indicate that as the feed temperature increases from 38 to 69 °C, the transmembrane flux increases from 2.7 to 16.3 kg/m^2^/h, indicating that temperature is the determining factor affecting the transmembrane flux of the fin tube gas–gap membrane distillation. As the driving force for mass transfer in membrane distillation is the difference in saturated vapor pressure on both sides of the membrane, the exponential increase in the value of the saturated vapor pressure of water with increasing feed temperature causes an increase in the difference in the saturated vapor pressure on both sides of the membrane, that is, an increase in the mass transfer driving force of the membrane distillation process, increasing the transmembrane flux. The theoretical value of *B* remains unchanged with temperature and is approximately 2.0 × 10^−7^ kg/m^2^/Pa/s. However, the experimental results show a slight decrease in B with increasing temperature due to the large volume of the feed pool, the higher temperature measurement of the feed, and the intensified thermal polarization phenomenon during membrane distillation.

Experimental results shown in Figure 3b indicate that as the mass concentration increases from 50 to 200 g/kg, the transmembrane flux decreases from 8.3 to 5.0 kg/m^2^/h. With the increase in feed concentration, the decrease in transmembrane flux and *B* value is observed, and crystallization occurs at some places on the membrane surface under high concentration conditions. This is because as the feed concentration increases, the saturated vapor pressure of the feed decreases, while the concentration polarization on the membrane surface increases. Additionally, the vapor pressure in the saline solution is related to the water activity. As the salt concentration increases, the water activity decreases and the vapor pressure decreases [31], causing a decrease in the transmembrane flux of the membrane distillation process due to changes in the density, viscosity, and diffusion coefficient of the feed [32]. Furthermore, the crystallization on the membrane surface may be another reason for the decrease in performance because it reduces the effective membrane surface available for evaporation. The concentration of the feed on the membrane surface may increase above the saturation concentration due to the permeation of water molecules, leading to crystallization. Moreover, due to the contact with the fin aluminum tube, the temperature of the membrane surface is lower than that of the feed, resulting in a decrease in the solubility of solutes and crystallization.

When the feed flow rate was increased from 100 to 500 mL/min, the transmembrane flux increased from 8.3 to 11.6 kg/m^2^/h, as shown in Figure 3c. As the feed flow rate increased, the transmembrane flux and *B* value of the gap–tube membrane distillation also increased. This is because the flow velocity of the feed passing through the membrane surface increases with the increase in feed flow rate, which disrupts the membrane surface boundary layer due to the disturbance, reducing the thermal resistance near the membrane surface and increasing the mass transfer driving force, resulting in an increase in the transmembrane flux. The experimental results show that the increase in transmembrane flux from 200 to 400 mL/min is only 13%, indicating that the flow rate is not a key factor affecting the performance of membrane distillation. The salt rejections of the membrane distillation tests are also larger than 99.9%.

### 3.3. Capacity Factor

**Figure 4:**
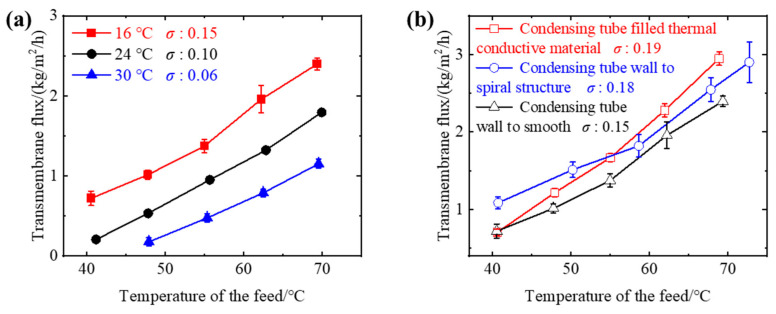
Functional performance of membrane distillation process under different cooling conditions. (**a**) Different cooling air temperatures and (**b**) internal structures of cooling channels (the temperatures of air were 16 ± 1 °C) in the tapered finned tubular air gap membrane distillation process. The feed flow rate was 100 mL/min. The concentration of the feed was 50 g/kg. The cooling medium was air with a flow rate of 2.8 m/s.

The feed side structure of the membrane distillate did not change, and the *B* value could be considered as unchanged to 2.0 × 10^−7^ kg/m^2^/Pa/s. To investigate the effect of cooling side parameters on the performance of membrane distillation, experiments were conducted by changing the air temperature and modifying the conditions inside the condensation tube, such as by turning the inner wall surface into a spiral structure and filling it with high thermal conductivity materials to increase the heat transfer area. As shown in Figure 4a, under the conditions of air cooling, the transmembrane flux of membrane distillation can reach a maximum of 2.5 kg/m^2^/h. Compared with the flux of water cooling, under the condition of air temperature at 24 ± 1 °C, the transmembrane flux can reach only 1/10 of the transmembrane flux under water cooling (cooling water temperature at 24 ± 1 °C), and the corresponding *σ* value is about 0.10, which is lower than the *σ* value of 0.45 in the forced convection air-cooled membrane distillation device constructed by Cong et.al [18]. This is because the air flow inside the condensation tube is almost zero for radiation heat transfer between the condensation wall surface and the large space, resulting in the *σ* value of the tubular membrane distillation being lower than that of the plate membrane distillation under the same conditions.

During air-cooled membrane distillation, the cooling air temperature significantly affects the *σ* value. As shown in Figure 4a, as the air temperature decreases, the transmembrane flux and *σ* value of the membrane distillation process can increase. When the cooling air temperature is lowered from 30 to 16 °C, the *σ* value can increase from 0.06 to 0.15. However, lowering the air temperature further requires more energy input. In order to reduce energy input, we increased the transmembrane flux and *σ* value by changing the structure of the condensation wall. As shown in Figure 4b, the spiral condensation wall with a higher surface area ratio is related to a larger *σ* value. Filling the thermal conductive material in the condensation tube of the finned tube can also improve heat transfer and increase the transmembrane flux and *σ* value of membrane distillation. The experimental results show that the transmembrane flux increased by 100% when the air temperature was lowered from 30 to 16 °C, and improving the morphology of the condensation wall can increase the transmembrane flux by 20%. Although the *σ* value did not reach 1, it verifies the applicability of air cooling to finned tube air gap membrane distillation and provides a simple method to predict the transmembrane flux of the membrane distillation process.

## 4. Conclusions

This study outlines the development of a finned tubular air gap membrane distillation configuration and examines the impact of air gap structure, feed parameters, and cooling conditions on membrane distillation performance. The study incorporates the parameters *B* and *σ* into the finned tubular air gap membrane distillation. Experimental findings reveal that the tapered finned tubular air gap membrane distillation device has the highest mass transfer capacity, and that the benefits of the tapered structure outweigh those of the increased condensation area. During the membrane distillation process, the temperature of the hot feed solution has a minimal effect on the *B* value. Increasing the concentration of the hot feed solution decreases the *B* value, while increasing the flow rate of the hot feed solution reduces polarization, and subsequently increases the *B* value. The maximum transmembrane flux obtained in the experiment is 16.3 kg/m^2^/h, indicating the excellent water-treatment capabilities of the finned tube air gap membrane distillation configuration. The efficiency coefficient of the air-cooled finned tubular membrane distillation device can reach 0.19, confirming the suitability of air cooling for the finned tubular air gap membrane distillation structure. By reducing the temperature of the cooling air and modifying the inner wall structure of the condensation surface to increase the surface area ratio, the efficiency coefficient of the membrane distillation process can be enhanced.

## Figures and Tables

**Table 1:** Parameters of the PTFE porous membranes.

Nominal Pore Size	Porosity	Thickness	Water Contact Angle
0.22 µm	75 ± 5%	35 ± 5 µm	152 ± 5°

**Table 2:** Parameters of the finned tubes.

Outer Diameter	Inner Diameter	Tube Length	Air Gap Width	Air Gap Depth
18 ± 0.3 mm	10 ± 0.3 mm	200 ± 0.3 mm	4 ± 0.3 mm	2 ± 0.3 mm

**Table 3:** Uncertainties of the instruments used in the membrane distillation tests.

K-Type Thermocouple	Peristaltic Pump	Electronic Balance	Digital Caliper
±0.1 °C	±0.1 mL/min	±0.01 g	±0.01 mm

**Table 4:** Comparison of transmembrane flux for different air gap membrane distillation modules.

Reference	Module	Air Gap Depth/mm	Salinity	Feed/Coolant Temperature/°C	Air Gap Structure	Flux /(kg/m^2^/h)
[14]	Plate and frame	3.2	35 g/kg	60/40	Plastic mesh	2.3
					Copper foam	5.0
[15]	Plate and frame	1	30,000 ppm	55/10	Circle fins	3.98
					Rectangle fins	4.11
					Triangle fins	3.92
[25]	Finned tubular	1	0.55%	75/50	4 Grooves	10.5
[26]	Cylindrical	3	5 g/L	60/30	-	3.6
[27]	Helical	3	30 g/L	55/29	-	4.35
This work	Finned tubular	2	50 g/kg	55/25	Tapered	8.21
					Flat	7.39
					Expanded	6.07

## Data Availability

The data presented in this study are available on request from the corresponding author.

## Nomenclature

*A*	Size of the test membrane (m^2^)
*B*	Total mass transfer coefficient (kg/m^2^/Pa/s)
*c_f_*	Concentration of the feed
*c_p_*	Concentration of the permeate water
*J*	Transmembrane flux (kg/m^2^/h)
*m*	Mass of the permeate water collected (kg)
*P_f_*	Vapor pressure of the feed side (Pa)
*P_c_*	Vapor pressure of the cooling side (Pa)
*R*	Salt rejection
*t*	Predetermined time of the experimental process (h)
Greek letter	
*Σ*	Efficiency coefficient
